# Investigation of antifungal and antibacterial potential of green extracts of propolis

**DOI:** 10.1038/s41598-024-64111-7

**Published:** 2024-06-13

**Authors:** Jeslin Cheruvathoor Jenny, Piotr Marek Kuś, Piotr Szweda

**Affiliations:** 1 Department of Pharmaceutical Technology and Biochemistry, Faculty of Chemistry, Gdańsk University of Technology, Ul. G. Narutowicza 11/12, 80-233 Gdańsk, Poland; 2 Department of Pharmacognosy and Herbal Medicines, Faculty of Pharmacy, Wroclaw Medical University, Ul. Borowska 211a, 50-556 Wrocław, Poland

**Keywords:** Propolis, Extraction, Natural deep eutectic solvents (NADES), Antimicrobial activity, Microbiology, Natural products

## Abstract

Propolis extracts have been used in traditional medicines since ages due to its advantageous complex chemical composition. However, the antibacterial and antifungal activity of poplar propolis extracts prepared in Natural Deep Eutectic Solvent (NADES) are seldom studied. This study investigates suitable alternate for ethanol as a solvent for extraction for Polish poplar propolis. It also attempts to identify suitable extraction condition for the efficient transfer of compounds from propolis to the solvents. The extraction efficiency of NADES extracts was assessed in terms of total phenolic content, antioxidant activity and antimicrobial activity. The chemical composition of the extracts was analysed using UHPLC-DAD-QqTOF-MS. Four extracts, prepared in Propylene Glycol, Choline Chloride:Propylene Glycol (1:3), Choline Chloride:Propylene Glycol (1:4) and Choline Chloride:Glycerol (1:2), demonstrated activity and properties similar to ethanolic extract and extraction at 50 °C was found the most suitable for propolis. HPLC analysis confirmed that the chemical cocktail extracted by these solvents from propolis were identical with minor variations in their concentration as compared to its ethanolic extract. Thus, extracts of propolis at 50 °C in Propylene Glycol, Choline Chloride:Propylene Glycol (1:3) and Choline Chloride:Propylene Glycol (1:4) can be alternates for ethanolic extracts.

## Introduction

Propolis is a plant-derived honeybee product used in traditional medicines since ages due to its complex therapeutically relevant chemical composition. Studies have established numerous health benefits like antimicrobial, antioxidant, anti-inflammatory, immunostimulant, hepatoprotective, anti-ulcer, antitumor and wound-healing properties of propolis^1–4^. Most of the bioactive molecules identified from it are secondary plant metabolites like phenolics and flavonoids like chrysin, galangin, pinocembrin, pinobanksin etc.^5^. Thus, the chemical composition of propolis obtained from different geographical area can vary according to the variation in local flora. In this study we focus on propolis collected in Poland (Europe). Typical propolis from temperate zones originates principally from different poplar and aspen buds exudates as well as birch buds. The native species in Poland, are black poplar (*Populus nigra* L.), European aspen* (Populus tremula* L.), white poplar (*Populus alba* L.), silver birch *(Betula pendula* Roth) and white birch (*Betula pubescens* Ehrh.)^6^. The chemical composition of natural products extracts and their derivatives depends on the solvent polarity and method of extraction^7,8^. Hydroethanolic solutions are the most common solvent for the extraction of propolis. However, there is a need for alternate formulations, as ethanolic extract induces irritability on skin due to its aggressive nature and is also unsuitable for children, pregnant and breastfeeding woman and some religious communities^9^. Propylene glycol (PG), polyethylene glycol (PEG), glycerol (Gly) and plant oils are a few alternative formulations to alcoholic solutions which are effective, safe and non-toxic in a pharmaceutical perspective^10–12^.

Another alternative extractants are Deep Eutectic Solvents (DES) that have gained popularity in the past decades, not only as a solvent for extraction but also in the fields of bio-catalysis, electrochemistry, CO_2_ capture and for various biomedical applications^13–15^. DES is a mixture of two or more components which form a single phase with a unique lowered melting point (lower than individual components) and essentially contains a hydrogen bond donors and acceptors. DES composed of primary metabolites are termed Natural DES/NADES^16^. The use of DES/NADES for natural product extraction aligns with green chemistry principles. The ability to adjust the solvent's polarity through DES composition enhances its extraction capabilities. While NADES has been explored for various natural product extractions, research on its effectiveness for extracting propolis is limited^17–20^.

In the current study we attempt to extract different propolis samples in 10 solvent systems in three different extraction conditions; at room temperature for 100 h (the traditional approach used by many beekeepers), at 50 °C for three hours and under ultrasonication for one hour. The solvents include 70% ethanol, neat propylene glycol and different NADES. All the extracts are evaluated for their antioxidant activity, total phenolic content, antibacterial, antifungal activity, and pH. Based on the results of these preliminary studies, five solvents and one method of extraction was finalised. The efficiency of extraction of the selected solvents was confirmed with two new samples of propolis; their antifungal and antibacterial activity were investigated. Further, HPLC analysis of extracts confirmed the extraction of all compounds from propolis in each solvent.

## Materials and methods

### Chemicals

Choline chloride, L-lysine, propylene glycol, glycerol, DL-lactic acid, citric acid, gallic acid, Na_2_CO_3_, 1,1-diphenyl-2- picrylhydrazyl radical (DPPH), Nile red, Folin-Ciocalteu reagent, ferric chloride, 2,4,6-Tri(2-pyridyl)-s-triazine (TPTZ), acetic acid, ferrous sulphate, resazurin, were purchased from Merck. Methanol and ethanol were purchased from POCH. Propolis samples were collected from various apiaries located near Gdansk, a city in northern Poland.

### Preparation of solvents

10 different solvents were used for the extraction of propolis which included neat propylene glycol, natural deep eutectic solvent (NADES) and 70% (v/v) hydroethanolic solution (as a reference). The composition of the solvent systems is given in Table 1. NADES were prepared by mixing the components in the prescribed ratios with the aid of a magnetic stirrer at 50 °C until a homogenous solution is formed.

**Table 1:** Composition of solvents used for extraction of propolis.

Components	Ratio
Propylene glycol (PG)	
Ethanol:water (EtOH)	7:3 (v/v)
l-lysine:water (Lys:H_2_O)	1:10 (w/v)
DL-lactic acid:water (LA:H_2_O)	85:15 (w/w)
Citric acid:propylene glycol (CA:PG)	1:4 (molar ratio)
Choline chloride:propylene glycol (CC:PG)	1:3 (molar ratio)
Choline chloride:propylene glycol (CC:PG)	1:4 (molar ratio)
Choline chloride:glycerol (CC:Gly)	1:2 (molar ratio)
Choline chloride:lactic acid:water (CC:LA:H_2_O)	1:1:1 (molar ratio)
Choline chloride:lactic acid:water (CC:LA:H_2_O)	1:2:2 (molar ratio)

### Polarity

Polarity of the prepared solvents was determined using the solvatochromic dye Nile red^21,22^. A 10 mM stock solution of the dye in ethanol was prepared and stored at 4 °C. 20 µL of this stock solution was added to 2 mL of NADES and mixed well in a shaker until a homogeneity is obtained. The absorbance was measured in a cuvette in a UV spectrophotometer in the range 400–700 nm. Pure NADES solvent was used as the blank. The solvent polarity was calculated based on Eq. (1) using the obtained λ_max_.

1 $${E_{NR} \left( {\frac{kcal}{{mol}}} \right) = \frac{hcN_A}{{\lambda \max }} = \frac{28,591}{{\lambda \max }}}$$

where, E_NR_ is the molar transition energy in kcal/mol, h is Plank’s constant, N_A_ is Avogadro number, c is the velocity of light and λ_max_ is wavelength in nm.

### Preparation of propolis extracts

Three samples of propolis labelled 1, 2, and 3 were frozen at − 80 °C for 2 h and ground into a fine powder. 0.5 g propolis was then added with 10 mL of solvent and extraction was performed in 3 different conditions (i) at room temperature with shaking (180 rpm) for 100 h (ii) ultrasonicated for 1 h and (iii) at 50 °C with shaking (180 rpm) for 3 h. Upon completion of the prescribed time the content was centrifuged at 10,000 rpm for 20 min to separate the extract from solid residue. The obtained extracts were collected after filtration to remove any solid residues and were stored in dark. The same procedure was adopted for two more samples labelled 4 and 5 which were extracted at 50 °C for 3 h.

### Total phenolic content

Total phenolic content of the propolis extract was estimated using the Folin-Ciocalteu method with slight modifications^23^. 100 μL Folin-Ciocalteu reagent was added with 20 μL propolis extract diluted 100 folds in ethanol. After 5 min of incubation 600 μL Na_2_CO_3_ (0.94 M) was added followed by ultrapure water to make up to a final volume of 2 mL. The mixture was incubated for 90 min at ambient temperature and absorbance of the samples were measured at 725 nm. The result was expressed as milligrams of gallic acid equivalence per gram of raw propolis. The experiment was conducted in triplicate.

### DPPH assay

The radical scavenging activity of the extracts was measured using DPPH assay following previously reported procedure^23^. The prepared extracts of propolis were diluted 10 times in methanol prior to the assay. A twofold serial dilution in methanol was adopted to yield a final concentration in a range between 5 and 0.00985% (v/v) and a final volume of 100 µL in a 96-well microtiter plate. 100 µL of 0.2 mM DPPH solution was added to each well with extracts and methanol (control) prepared in triplicate. The microtiter plates were incubated for 30 min in dark at ambient temperature and absorbance was measured at 517 nm. The % of radical scavenging activity was calculated from Eq. (2) from the values obtained for absorbance (*A*_*DPPH*,_ *A*_*MeOH*,_ *A*_*Sample*_). The IC_50_ value was calculated from % of radical scavenging activity using GraphPad Prism 5.

2 $${\text{\% of Radical Scavenging Activity}} = 100 \times \frac{{\left( {A_{DPPH} - A_{MeOH} } \right) - \left( {A_{Sample} - A_{MeOH} } \right)}}{{\left( {A_{DPPH} - A_{MeOH} } \right)}}$$

where, A_DPPH,_ A_MeOH_ and A_Sample_ are the values of absorbance measured for of DPPH solution, methanol and sample respectively.

### FRAP assay

Antioxidant activity of propolis extract was also assessed using FRAP assay^24^ with minor changes. In a 96 well microtiter plate 190 µL of FRAP reagent was added with 10 µL propolis extract diluted 50 folds in ethanol and incubated in dark for 30 min. A calibration curve was plotted with 0.5–8 mM solution of Fe_2_SO_4_ in acetate buffer. The experiment was conducted in triplicate. The absorbance was measured on a microtiter plate reader at 593 nm.

### Determination of minimum inhibitory concentration (MIC)

The antimicrobial activity of all the extracts were tested against gram positive bacteria *Staphylococcus aureus* ATCC 29213, *Staphylococcus aureus* ATCC 25923 and four strains of pathogenic fungi of the genus *Candida* spp., namely: *Candida albicans* SC5314, *Candida glabrata* DSM 11226, *Candida krusei* DSM 6128 and *Candida parapsilosis* DSM 5784. Serial two-fold broth microdilution method was adopted to investigate the minimum inhibitory concentration. An overnight culture of the cells was used for the preparation of the cell suspension in phosphate buffer solution. The optical density was adjusted to 0.1 at 600 nm for bacterial strains and to 0.1 at 660 nm for the selected fungi variants. The suspensions were diluted 1/100 (v/v) in MHB2 for bacteria and 1/50 (v/v) in RPMI 1640 medium supplemented with MOPS 3.5% (w/v) and 2% (w/v) glucose (pH of the medium was adjusted to 7.0 with solid NaOH) for fungi, to obtain a final cell concentration of approximately 10^6^ CFU/mL (in the case of staphylococci) and 2 × 10^4^ CFU/mL (for yeasts strains). Two-fold serial dilution of propolis extracts in respective mediums in a 96-well microtiter plate followed by the addition of 100 µL of the diluted cell suspensions resulted in final propolis concentration varying between 2.5 and 0.08% (v/v). The plates were incubated at 37 °C for 24 h. The minimum inhibitory concentration (MIC) was estimated by optical density measurements of the plates at 531 nm and confirmed by resazurin test^25^.

### pH measurements

pH of the extracts at a final concentration 2.5% (v/v) of solvent in MHB2 medium and RPMI 1640 supplemented with MOPS and 2% glucose was measured using a pH meter (Mettler Toledo).

### Kill-time assay

Time-kill assay was performed with the most active sample of propolis (sample number 4) against *C. glabrata* DSM 11226 and *S. aureus* ATCC 25923*.* An overnight culture of the cells was suspended in sterile water with optical density adjusted to 0.1 at 660 nm and 600 nm for yeasts and staphylococci respectively. Subsequently 1 mL of each suspension was diluted 100 folds in appropriate medium RPMI for *C. glabrata* and (final cells concentration was approximately 10^4^ CFU/mL) and MHB2 for bacteria (final cells concentration was approximately 10^6^ CFU/mL). The prepared inoculum was divided into five parts of equal volume and was added with 4xMIC of the extracts of the most active sample of propolis (sample 4) and was incubated at 37 °C. 50 μL of each part with appropriate dilutions were spread on a SAB agar plate (*C. glabrata*) and a Baird-Parker agar plate supplemented with egg yolk tellurite emulsion (*S. aureus*) at time 0, 2, 5 and 24 h. The plates were incubated overnight and the number of colonies were counted.

### UHPLC-DAD-QqTOF-MS analysis

Acetonitrile (both gradient grade and LC/MS grade) and LC/MS grade water as well as formic acid was purchased from Sigma-Aldrich. Analytical standards of caffeic acid, *p*-coumaric acid, ferulic acid, isoferulic acid, vanillin, pinobanksin, apigenin, kaempferol, chrysin, pinocembrin, sakuranetin, galangin, pinostrobin, pinocembrin chalcone, pinocembrin dihydrochalcone were purchased from Extrasynthese. Ultrapure water (< 0.06 μS/cm) was obtained from Hydrolab HLP20UV (Hydrolab, Straszyn, Poland) purification system. For UHPLC analyses, Thermo Scientific™ UltiMate™ 3000 system (Thermo Scientific™ Dionex™, Sunnyvale, CA, USA) with an autosampler and DAD detector and Compact QqTOF-MS detector (Bruker, Darmstadt, Germany) was applied. Chromatographic separation was performed on Kinetex® C18 polar 2.6 µm, 100 Å, 150 × 2.1 mm analytical column with guard-column (Phenomenex, Torrence, CA, USA) thermostated at 20 ± 1 °C. The injection volume of the sample was set to 1 μL. The mobile phase used for chromatographic separation consisted of 0.1% formic acid solutions in water (solvent A) and acetonitrile (solvent B). The flow rate was set at 0.4 mL/min and the separation was obtained using following gradient: 95% of solvent A isocratic for 10 min, decreasing to 80% within 1 min and held isocratic for another 10 min, decreasing to 65% A within 1 min and held isocratic for 8 min, decreasing to reach 40% A within 15 min and isocratic for another 15 min. Subsequently, the elution solvent increased 100% B, the column was rinsed and then the solvent returned to 95% A. Before the next analysis the system was stabilized. Spectral data was recorded in the 200–600 nm range as well as at 280, 320, 360 nm. MS detector was used in ESI negative mode, ion source temperature was set at 100 °C, nebulizer gas pressure at 2.0 bar, dry gas flow 0.8 L/min and temperature 210 °C. The capillary voltage was set at 2.20 kV and collision energy at 8.0 eV. Internal calibration was obtained by injection of 10 mM solution of sodium formate clusters. For ESI–MS/MS experiments, collision energy was set at 35 eV and nitrogen was used as collision gas. Before the analysis, all the extracts were filtered through CHROMAFIL® 0.2 µm, Ø13mm, H-PTFE syringe filter (Macherey–Nagel, Düren, Germany). Standard solutions were prepared in ethanol and the working standard solutions were diluted in ethanol or in ultrapure water. The calibrations curves were prepared in concentration range concentrations of 6.25–200 µg/mL and the correlation values were 0.9996–1.0000. The content of derivatives of caffeic acid, *p*-coumaric and pinobanksin were calculated as their equivalents similarly as chalcones, that were calculated as equivalents of pinocembrin chalcone or pinocembrin dihydrochalcone. Additionally, the results were corrected taking into account differences in molecular mass.

## Results and discussion

### Polarity of solvents

Polarity of the solvent used for extraction qualitatively and quantitatively determines the extraction efficiency. More polar solvents can extract more polar compounds from propolis. The polarity of the NADES prepared are listed in Table 2. The solvatochromic dye failed to dissolve in Lys:H_2_O. Hence the polarity of only seven NADES could be estimated. The polarity of all the solvents were similar to 70% EtOH. A lower E_NR_ value corresponds to a higher polarity and vice versa, making LA:H_2_O and CA:PG the most polar solvents. Thus, minor variations in the chemical composition and activity of NADES extracts can be anticipated against their alcoholic reference.

**Table 2:** Polarity of the prepared solvents.

Solvent	E_NR_ [kcal/mol]
70% EtOH	50.60
LA:H_2_O	47.81
CA:PG (1:4)	47.81
CC:PG (1:3)	50.78
CC:PG (1:4)	50.69
CC:Gly (1:2)	49.98
CC:LA:H_2_O (1:1:1)	48.21
CC:LA:H_2_O (1:2:2)	47.89

### Preliminary analysis

A preliminary study was conducted with three samples of propolis labelled 1, 2, and 3, to determine the solvents suitable for extraction which can exhibit comparable chemical and biological activity to ethanolic extract. It is also an attempt to identify the most suitable mode of extraction for efficient transfer of phenolics from propolis to the solvent regardless of its viscosity. All the solvents used, except Lys:H_2_O and 70% ethanol, are highly viscous liquids at room temperature. Extractions assisted by ultrasonication or elevated temperatures are advantageous over the conventional method, particularly for viscous deep eutectic solvents. Temperature and viscosity are factors inversely proportional to each other and result in efficient transfer of mass from propolis to the NADES. On the other hand, ultrasound waves produce cavitation bubbles in the solvents leading to increased yield of extraction. However, higher temperature can lead to degradation of polyphenols, and ultrasonication poses the risk of free radical generation^26^. Thus, using three different methods of extraction can aid in quantitative and qualitative evaluation of the extraction efficacy. It was observed that the extracts of each sample of propolis in different solvents appeared in a spectrum of colours ranging from yellow to brownish black, which can be an indicator of their varying composition and interactions with solvent.

### Total phenolic content

**Figure 1:**
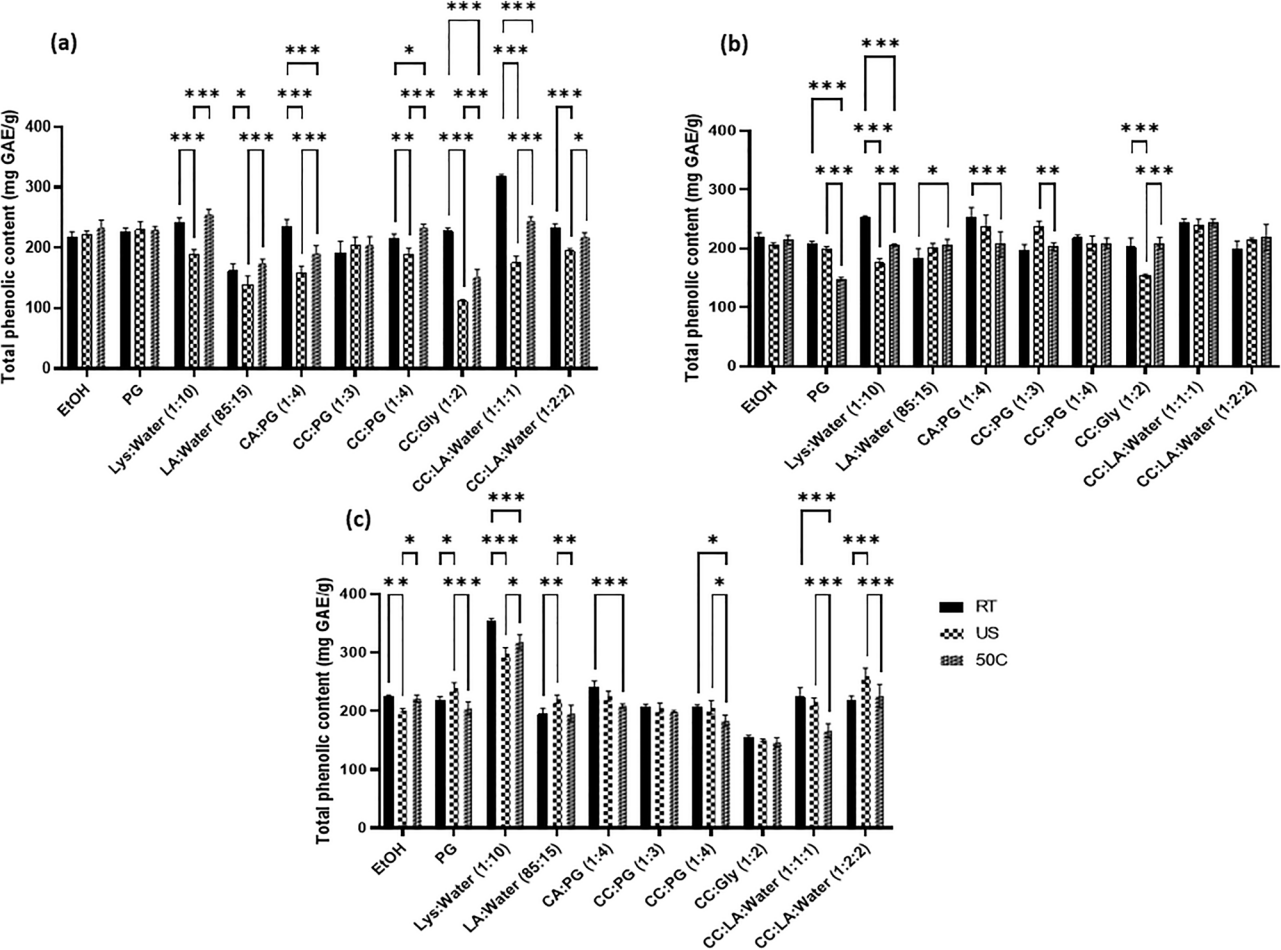
Total phenolic content of propolis 1 (**a**), 2 (**b**) and 3 (**c**) shows the phenolic content quantified as mg of GAE/g of raw propolis at room temperature (RT), ultrasonication (US) and 50 °C (50 °C) represented as TPC ± SD. P < 0.332 (*), P, 0.002 (**), P < 0.001 (***).

Phenolic compounds reduce the phosphomolybdic–phosphotungstic acid present in the Folin-Ciocalteu reagent to form a blue complex whose intensity is dependent on the concentration of phenolics^27^. The total phenolic content (TPC) of the samples 1, 2 and 3 varied with solvents and extraction conditions. Ethanolic extract of each sample is taken as the reference for the evaluation of TPC for specific extraction conditions. The TPC of the samples 1, 2 and 3 are shown in Fig. 1. It is observed that the amount of phenolics extracted in ethanol is unaffected by the method of extraction (statistically important differences were noted only for propolis 3, but even in the case of this product, the absolute values of phenolics content in extracts obtained using different methods differed slightly—less than 10%). While the method of extraction significantly impacted effectiveness of recovery of phenolics in some other solvents systems, e.g. in the case of propolis sample 1 statistically important differences were observed for all NADES except of CC:PG(1:3). On the other hand, the composition of the solvents also importantly affected the efficiency of phenolics extraction when the same method of extraction process was applied. For about 50% difference in TPC was observed for the extracts of sample 1 obtained with the classical approach (room temperature for 100 h) in 70% (v/v) ethanol and CC:LA:Water (1:1:1). Concluding results of this part of our study, it is revealed that PG and other NADES solvents extracted sufficient amount of phenolics, comparable with the reference ethanol extract. Lys:H_2_O demonstrated a superior extraction efficiency for sample 3 over others (independent of the method of extraction). However, no method of extraction guaranteed consistent high yield of phenolics in all three tested samples of propolis.

### Antioxidant activity

**Figure 2:**
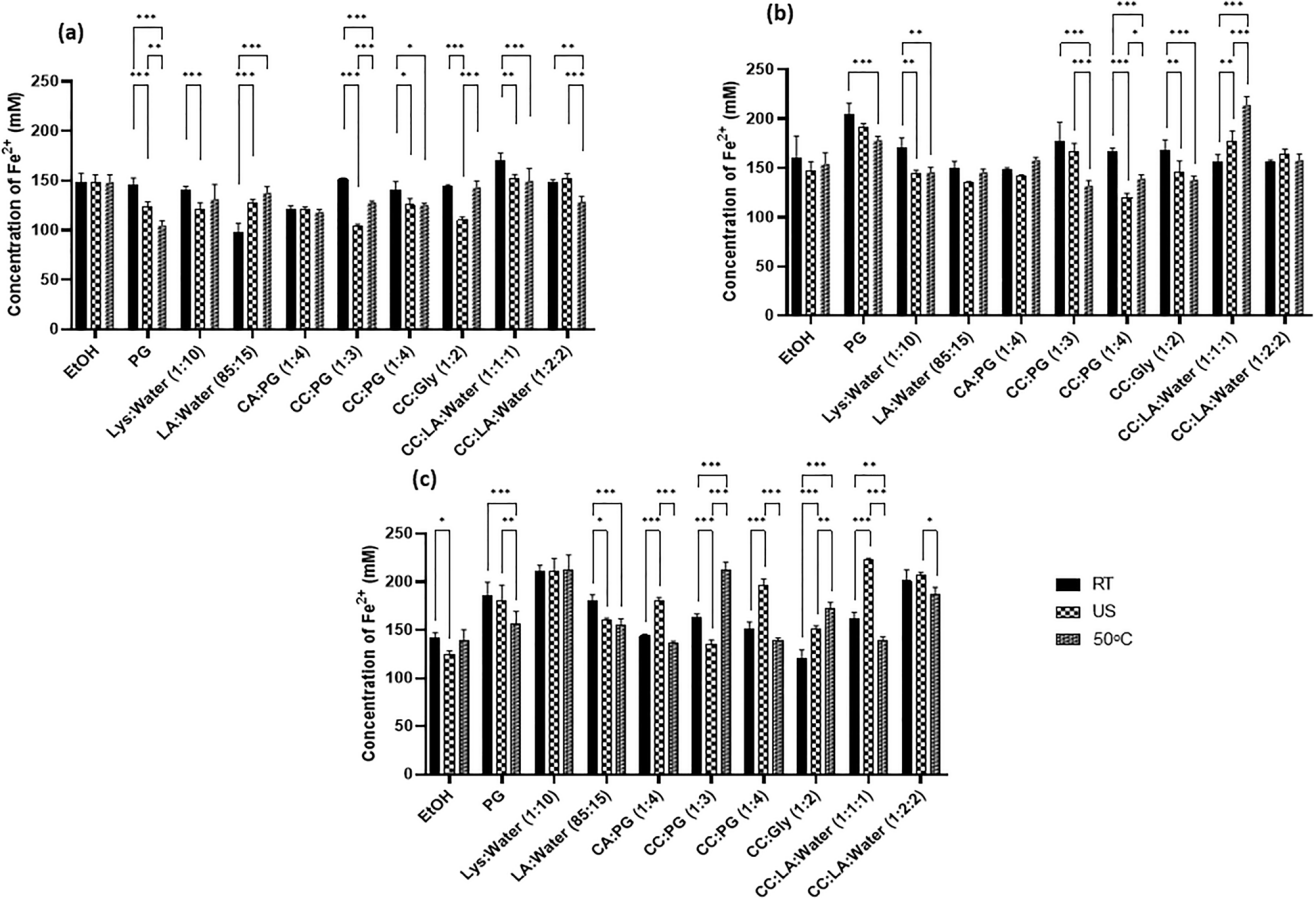
Ferric reducing power of propolis (**a**–**c**) shows the phenolic content quantified as mM of Fe^2+^ at room temperature (RT), ultrasonication (US) and 50 °C (50 °C) represented as mM Fe^2+^ ± SD, P < 0.332 (*), P, 0.002 (**), P < 0.001 (***).

Oxidation processes have different mechanisms, demanding more than one method for a better understanding, herein we assess the antioxidant activity by FRAP assay and DPPH assay. The FRAP assay is a widely used method to understand the antioxidant potential. However, all the compounds responsible for the conversion of Fe^3+^ to Fe^2+^ are not necessarily antioxidants^28^. Thus, FRAP assay along with DPPH assay were used and the results are summarised in Figs. 2 and S1. DPPH assay gives a quantitative measure of the total antioxidant potential. However, analysis of the results of this assay (Fig. S1) led to general conclusion that this method is not suitable for the determination of the antioxidant potential of propolis extracts produced with NADES. Mainly because of observed huge differences in the values of antioxidant potential of extracts produced with different solvents, and also (which is even more important) extracts obtained in the same solvent but using different methods of extraction. In some cases these differences were at the level 400 or even 600%, e.g. extracts of propolis 3 produced in CA:PG (1:4) or LA:Water (85:15). These differences completely do not correspond to the results of the assays aiming in determination of the total phenolics content and also FRAP method. The appropriate control measurements revealed that the solvents used for extraction alone do not affect the results of the assay and no important differences in DPPH reducing potential of ethanol extracts produced with different methods were observed. This all together can suggest that other than phenolics, ingredients affecting DPPH, were extracted from raw material samples with different solvents and/or under different conditions of the process of extraction. A bit better results were obtained for FRAP method, particularly for samples 1 and 2 (Fig. 2a,b) which showed results comparable to its alcoholic counterpart. Figure 2c indicates that most extracts of sample 3 generally showed a superior antioxidant activity than their corresponding ethanolic extract and some important differences are noted for extracts obtained in the same solvent but with different methods. This observation also suggests that some other compounds/antioxidants, not only phenolics, are recovered from the raw materials and propolis number 3 contains the largest amount of these ingredients. However, it is well proven that the health-promoting potential of propolis results mainly from the content of phenolic compounds, so in our opinion determination of antioxidant potential (either with FRAP or DPPH method) should not be recommended for indirect assessment of the health-beneficial potential of NADES extracts of propolis.

### Antimicrobial activity

#### Determination of minimum inhibitory concentration against *C. albicans*

MIC values against *C. albicans* are listed in Table 3. The extracts produced from propolis sample assigned with number 1 exhibited the highest antifungal potential. Interestingly, the PG extract showed similar antifungal (in two cases slightly higher) effectiveness in inhibition of *C. albicans* growth as the reference extracts produced with 70% (v/v) ethanol. However, from the point of view of the main goal of this study—assessment of possibilities of using NADES for preparing propolis extracts, the most important observation is that the extracts prepared with five out of eight NADES, namely LA:H_2_O, CA:PG, CC:PG (1:3), CC:PG (1:4) and CC:LA:H_2_O (1:1:1) also showed satisfactory, usually only twice lower, antifungal activity compared to reference hydroalhoholic extract. In our opinion these formulations of propolis can be also considered for the treatment of skin and mucous membranes candidiasis. Despite their lower activity, they are free from other disadvantages of alcohol extracts of propolis, of which the irritating properties and burning taste are the most important. The extracts prepared with three other solvents, particularly Lys:H_2_O exhibited lower antifungal potential. MIC assay when performed with pure solvents indicated no inhibition in the growth of *C. albicans* leading to the conclusion that the antifungal activity of the extracts in all the solvents are exclusively from the compounds extracted from propolis.

**Table 3:** MIC values in % (v/v) of propolis extracts (samples 1, 2 and 3) obtained at different conditions (*RT* -room temperature, *US* -ultrasonication, *50 °C* -elevated temperature) against *C. albicans.*

Solvent	1 RT	1 US	1 50 °C	2 RT	2 US	2 50 °C	3 RT	3 US	3 50 °C
EtOH	0.3125	0.625	0.3125	0.625	0.625	0.625	1.25	0.625	0.625
PG	0.3125	0.3125	0.625	0.625	0.625	0.625	0.625	0.625	0.625
Lys:Water (1:10)	2.5	2.5	2.5	> 2.5	2.5	> 2.5	0.625	1.25	1.25
LA:Water (85:15)	1.25	0.625	0.625	1.25	2.5	1.25	1.25	1.25	1.25
CA:PG (1:4)	0.625	1.25	0.625	1.25	2.5	1.25	1.25	2.5	2.5
CC:PG (1:3)	0.625	0.625	0.625	1.25	2.5	1.25	1.25	1.25	2.5
CC:PG (1:4)	0.625	0.625	0.625	1.25	1.25	1.25	1.25	1.25	2.5
CC:Gly (1:2)	0.625	1.25	1.25	2.5	2.5	1.25	2.5	2.5	2.5
CC:LA:water (1:1:1)	0.625	0.625	0.625	1.25	1.25	1.25	1.25	1.25	1.25
CC:LA:water (1:2:2)	1.25	0.625	0.625	2.5	2.5	1.25	2.5	1.25	2.5

#### Determination of minimum inhibitory concentration against *S. aureus*

Propolis extracts are known for its activity against gram positive bacteria. The MIC of all the extracts against *S. aureus* ATCC reference strain is listed in Table 4. The results are expressed as v/v %, which predominantly varied between 0.625 and 0.156%. All the extracts showed MIC values comparable with alcoholic extract except Lys:water extract. CC:Gly was the least active amongst the solvents which can be attributed to its high viscosity. However, in contrary to *C. albicans* it was noticed that some of the prepared solvents (containing lactic or citric acid) also exhibited antistaphylococcal activity, with MIC values from 0.312 to 0.625% (v/v). This result can be explained by the fact that these solvents importantly affect acidity of the MHB medium (Table 4). Previous studies have proved that *S. aureus* is sensitive to pH^29^. Thus, the inhibition caused by extracts prepared in lactic acid and citric acid can partly be an attribute of the pH alteration they cause.

**Table 4:** MIC values in % (v/v) of propolis extracts (samples 1, 2 and 3) obtained at different conditions (*RT* -room temperature, *US* -ultrasonication, 50 °C -elevated temperature) against *S. aureus.*

Sample	1 RT	1 US	1 50 °C	2 RT	2 US	2 50 °C	3 RT	3 US	3 50 °C
EtOH	0.3125	0.3125	0.3125	0.625	0.625	0.625	0.625	0.625	0.625
PG	0.3125	0.3125	0.625	0.3125	0.3125	0.3125	0.3125	0.625	0.3125
Lys:water (1:10)	0.15625	0.625	0.625	> 2.5	> 2.5	> 2.5	0.15625	1.25	2.5
LA:water (85:15)	0.15625	0.15625	0.15625	0.3125	0.3125	0.3125	0.3125	0.15625	0.3125
CA:PG (1:4)	0.3125	0.15625	0.15625	0.15625	0.3125	0.3125	0.15625	0.3125	0.15625
CC:PG (1:3)	0.3125	0.3125	0.3125	0.625	0.3125	0.3125	0.3125	0.3125	0.3125
CC:PG (1:4)	0.3125	0.3125	0.3125	0.3125	0.15625	0.15625	0.3125	0.3125	0.3125
CC:Gly (1:2)	0.3125	0.625	0.625	0.625	0.625	0.625	0.625	0.625	1.25
CC:LA:water (1:1:1)	0.3125	0.3125	0.3125	0.3125	0.3125	0.3125	0.3125	0.3125	0.3125
CC:LA:water (1:2:2)	0.3125	0.3125	0.3125	0.3125	0.625	0.625	0.3125	0.3125	0.3125

### pH and stability

pH of the bacterial and fungal culture medium after addition of the solvent (final concentration 2.5% v/v) is listed in Table 5. All the solvents containing lactic acid and citric acids showed significantly low pH, making them extremely acidic for practical application. An attempt to increase their pH requires dilutions to an extent that will surpass the MIC of extracts produced in these solvents. Thus, solvents which do not interrupt the pH are selected for further studies. Another key observation was regarding the stability of the extracts. The extracts prepared in LA:H_2_O (all extraction condition) and ultrasonicated extracts in CC:LA:H2O (1:1:1), CC:LA:H2O (1:2:2), CC:PG (1:3) and CC:PG (1:4) of all propolis samples developed turbidity or precipitates within an observation period of two months. On the other hand, extracts prepared in room temperature and at 50 °C were more resistant to precipitation. The phenolic content in the extracts prepared in 70% EtOH, PG, CC:PG(1:3), CC:PG(1:4) and CC:Gly(1:2) in room temperature and elevated temperature is comparable. However, extraction at 50 °C can be considered superior to the other when the time of extraction is accounted. Further, the effect of temperature can be utilised to reduce the viscosity of solvents for better extraction.

**Table 5:** pH of solvents in culture medium.

Solvents	pH of solvent in MHB 2 medium	pH of solvent in RPMI medium
EtOH	7.11	6.9
PG	7.0	6.9
Lys:Water (1:10)	8.7	7.3
LA:Water (85:15)	2.1	2.3
CA:PG (1:4)	2.35	2.6
CC:PG (1:3)	6.9	6.9
CC:PG (1:4)	7.0	6.9
CC:Gly (1:2)	6.9	6.9
CC:LA:Water (1:1:1)	2.4	2.9
CC:LA:Water (1:2:2)	2.4	2.7

### Evaluation of samples 4 and 5

Based on the preliminary screening of extraction method and solvent to be used, extractions at 50 °C for 3 h in five solvents systems—(i)70% EtOH, (ii) PG, (iii) CC:PG (1:3), (iv) CC:PG (1:4) and (v) CC:Gly (1:2) was finalised to be used for the extraction of two more samples of propolis labelled 4 and 5. This part of research was performed to finally confirm possibility of preparing alternative formulations of propolis with selected NADES that exhibit antimicrobial potential (both antibacterial and antifungal) comparable to hydroalcoholic extracts. Moreover, we decided to determine antimicrobial activity of the extracts against other pathogenic species of *Candida* spp., namely: *C. glabrata*, *C. krusei* and *C. parapsilosis*. Antibacterial activity was also investigated with one additional reference *S. aureus* strain. Unfortunately, we were very limited with the amount of the propolis samples 1–3. Thus, we decided to perform this part of the project with two additional samples (assigned as 4 and 5) of propolis. The total phenolic content, antioxidant potential and antimicrobial activity of these extracts were compared with its alcoholic extract and HPLC analysis was performed to identify its chemical composition.

### Total phenolic content and antioxidant activity

It is reported that NADES essentially extracts all the compounds present in 70% ethanolic extract in different proportion if they are of similar polarity^30^. The total phenolic content of sample 4 and 5 are tabulated in Table 6 and varied between 265.30 ± 9.94 mg GAE/g to 210.46 ± 21.9 mg GAE/g and 314.17 ± 9.12 mg GAE/g to 210.46 ± 6.36 mg GAE/g respectively. PG and CC:PG(1:3) showed better efficiency of extraction of phenolics in sample 5 than its alcoholic counterpart while they showed the least efficiency in sample 4. CC:Gly which generally exhibited lower phenolic content maintained the trend in sample 5 while it proved to be better than its PG and CC:PG counterparts in sample 4. CC:Gly is the most viscous solvent among the used solvents, justifying its lower extraction efficacy. A similar effect of viscosity was demonstrated by CC:PG (1:3) and CC:Gly against *Sideritis scardica*, and *Plantago major,* two medicinal plants^22^. A lower extraction efficiency of CC:Gly was also observed for propolis obtained from Bulgaria extracted by ultrasonication^30^. Another study conducted by Tzani et al. quantified a lower amount of phenolics from Greek propolis extracted in different NADES by ultrasonication^31^. Further, a study conducted by Alpat and co-workers identified NADES, which extracted upto 65% of phenolics from propolis when compared to its 80% ethanolic extract^20^.

**Table 6:** Total phenolic content and ferric reducing powerof propolis extracts (samples 4 and 5) expressed as mg of GAE/g of propolis and mM Fe^2+^ respectively.

Sample	Total phenolic content		FRAP assay	
	4	5	4	5
EtOH	265.30 ± 9.94	301.06 ± 8.04	323.46 ± 12.94	364.14 ± 3.22
PG	210.46 ± 21.9	314.17 ± 9.12	268.50 ± 10.16	294.72 ± 3.89
CC:PG (1:3)	209.86 ± 21.27	304.04 ± 8.43	296.38 ± 2.39	303.15 ± 10.21
CC:PG (1:4)	233.71 ± 31.07	276.02 ± 0.85	276.77 ± 17.82	295.45 ± 7.00
CC:Gly	238.47 ± 5.9	210.46 ± 6.36	237.92 ± 5.43	216.14 ± 11.17

Because of the previously mentioned disadvantages of the DPPH assay the antioxidant potential of the extracts of propolis samples 4 and 5 was carried out only using the FRAP method and phenolics concentration was determined with the Folin-Ciocalteu reagent. The results are listed in Table 6. The first important observation from this part of the study is the fact that the FRAP assay demonstrated superior antioxidant activity of ethanolic extract, whilst PG and CC:PG (1:3) extracts of sample 5 contained slightly higher concentrations of phenolics compared to hydroalcoholic extract. Considering the extracts prepared in particular solvents the products containing larger concentrations of phenolics exhibit higher antioxidant potential. However, in the case of CC:PG (1:3) the proportions are slightly different than in the case of other solvents.

### Antimicrobial activity

Previous studies have shown that propolis inhibits the filamentation of *C. albicans*^32,33^ which can reduce the virulence potential and reduce the formation of biofilms. Polyphenols in propolis can interrupt the cell wall formation of fungi by forming complexes with proteins in the cell wall involved in chitin synthesis^34^. This theory was later confirmed^35^. Antifungal activity of propolis examined in this study is assessed by estimating the MIC value of each extract against *C. albicans, C. glabrata, C. krusei and C. parapsilosis* reference strains and the results are tabulated in Table 7. Both the sets of extracts were more effective against *C. glabrata, C. krusei and C. parapsilosis* as compared to *C. albicans*. Despite the higher phenolic content of extracts of sample 5, it showed lower antifungal activity when compared to sample 4. The results indicate that all these extracts can act as an alternate for ethanolic extract against the tested species as they exhibit comparable (maximum two times higher value of MIC) activity. However, the extracts produced with CC:Gly exhibit a bit lower antifungal potential.

**Table 7:** MIC values in % (v/v) of propolis propolis extracts (samples 4 and 5) against *Candida* spp. and *S. aureus.*

Sample	Strain tested					
	*C. albicans* SC5314	*C. glabrata* DSM11226	*C. krusei* DSM6128	*C. parapsilosis* DSM5784	*S. aureus* ATCC *29213*	*S. aureus ATCC 25923*
4 EtOH	0.3125	0.3125	0.15625	0.15625	0.3125	0.3125
4 PG	0.625	0.15625	0.15625	0.15625	0.3125	0.15625
4 CC:PG (1:3)	0.625	0.15625	0.15625	0.15625	0.3125	0.3125
4 CC:PG (1:4)	0.3125	0.15625	0.15625	0.15625	0.3125	0.3125
4 CC:Gly	0.625	0.3125	0.3125	0.3125	0.625	0.3125
5 EtOH	0.625	0.3125	0.3125	0.3125	0.625	0.625
5 PG	0.625	0.3125	0.3125	0.3125	0.625	0.625
5 CC:PG (1:3)	0.625	0.3125	0.3125	0.3125	0.625	0.625
5 CC:PG (1:4)	0.625	0.3125	0.3125	0.3125	0.625	0.625
5 CC:Gly	1.25	0.3125	0.625	0.3125	1.25	1.25

Several mechanisms of antibacterial activity of propolis are identified like inhibition of (i) synthesis of nucleic acid, (ii) energy metabolism, (iii) cell membrane proteins; alteration of (i) function of cytoplasmic membrane, (ii) ability to form biofilms, (iii) membrane permeability; and depressing bacterial resistance^36^. But the mechanistic action depends on both the antibacterial component and bacterial strain. The MIC values denoting the antibacterial activity tested against *S. aureus* ATCC 29213 and *S.aureus* ATCC 25923 is given in Table 7. All extracts of 4 and 5 showed the same MIC value excluding CC:Gly which required twice the concentration of other extracts to attain MIC against *S. aureus* ATCC 29213. The same trend was observed for extracts of sample 5 against *S. aureus* ATCC 25923 and agrees with the results obtained for *Candida* spp. (presented above) and also presented by the study conducted by Trusheva et al. PG showed the least MIC against *S. aureus* ATCC 25923*.* Very few studies have focussed on the antibacterial activity of NADES extracts of propolis. Hence it can be concluded the used solvents can be a suitable alternative for ethanolic extract against the given bacterial strains.

### Kill time assay

**Figure 3:**
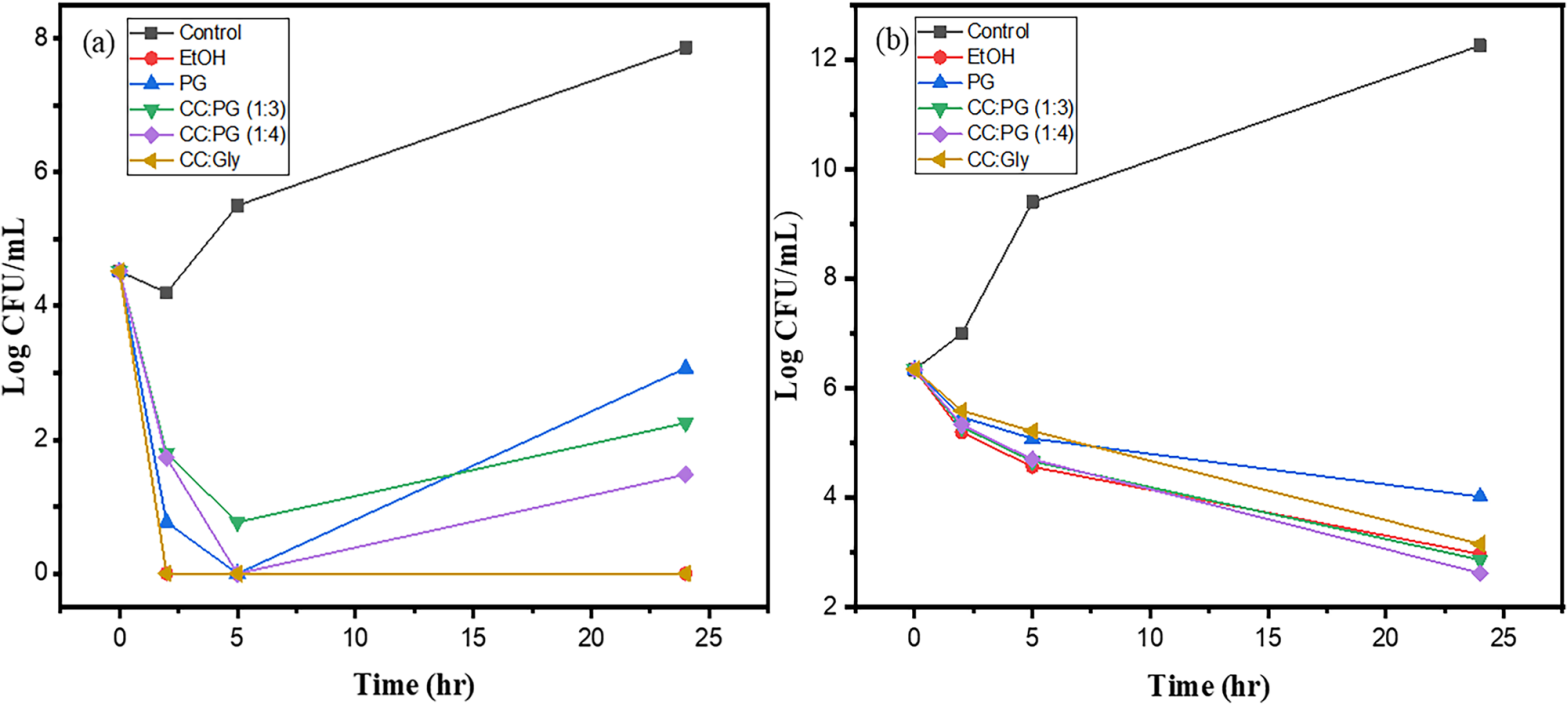
Kill time assay of extracts of propolis sample 4 against (**a**) *C. glabrata* DSM 11226 (**b**) *S. aureus* ATCC 25923. The results are shown as mean ± SD. Data point with no error bars are indicative of negligible value of SD.

Kill time assay of all 5 extracts of sample 4 was performed at a concentration of 4xMIC against *C. glabrata* and *S. aureus ATCC 25923* (Fig. 3). The results indicate the fungicidal activity of ethanolic extract and CC:Gly extract and growth inhibition by PG and both CC:PG extracts. An increase in cell viability of *C.glabrata* was observed in the PG and CC:PG extracts after 5 h, until when the growth rate reduced. The extract produced with CC: Gly combination as a solvent exhibited the highest MIC value. Thus, in the case of the kill time assay, this product was used at two times higher concentration compared to other extracts (in each case it was 4xMIC), which partly can explain observed differences in antifungal activity against *Candida* spp. strains tested. Results indicate that the antimicrobial activity of propolis extract against *S.aureus* ATCC 25923 is primarily growth inhibition at a concentration of 4xMIC. This observation is well in agreement with previous studies wherein ethanolic extracts of propolis were tested against the same strain^23^. CC:PG (1:4) extracts showed better inhibition than ethanolic extract followed by CC:Gly and PG extract. However, the differences were not large.

### HPLC analysis of the extracts of propolis samples 4 and 5

A total of 32 major compounds were identified in the propolis extracts by UHPLC-DAD-QqTOF-MS and quantified by UHPLC-DAD (Table 8, Fig. S2—Supplementary materials). The comparison of chromatographic profiles of different extracts is presented in Fig. S2. The chemical profiles of the samples were characterized by a variety of flavonoids (flavanones: pinocembrin, pinostrobin, sakuranetin; flavanonols: pinobanksin; flavones: apigenin, chrysin; flavonols: galangin, kampferol). Other relevant compounds included phenylpropanoid derivatives, mostly *p*-coumaric, caffeic, ferulic, isoferulic acids and their esters. The compounds were identified based on exact mass, MS^2^ fragmentation and UV spectra as well as reference compounds. Determination of phenylpropanoid derivatives was confirmed by the presence of appropriate losses and diagnostic ions (*p*-coumaroyl: 163/145, caffeoyl: 179/161, feruloyl: 193/175) in MS^2^ spectra of their esters. Based on the occurrence of specific components it is possible to confirm the probable botanical origin of the analyzed propolis samples. Both samples contained relevant amounts of phenylopropanoid glycerides (1,3-di-*p*-coumaroylglycerol, 2-acetyl-1,3-di-*p*-coumaroylglycerol, 2-acetyl-3-*p*-coumaroyl-1-feruloylglycerol) characteristic for aspen bud exudates^6,37^. On the other hand, the investigated samples contained also methylbutenyl esters of caffeic acids and caffeic acid phenethyl ester (CAPE) along with pinocembrin, chrysin, pinobanksin and pinobanksin acetate, characteristic *i.a.* for black poplar (*P. nigra*) and not present in aspen^6,37^. Occurrence of sakuranetin in the investigated samples may be related to black poplar, aspen or white birch, however, its high abundance suggests, it is related rather to white birch (*B. pendula*). It may be confirmed also by the presence of some amount of compounds characterized by pseudomolecular ions [M−H]^−^ peaks at m/z 365.2114 fragmenting (MS^2^) to 162(163)/145/118 (*p*-coumarate) which may be tentatively attributed to hydroxyl-β-caryophyllene *p*-coumarates characteristic for white birch buds^6,37^.

**Table 8:** The content of major compounds in different propolis extracts determined using UHPLC-DAD (data are expressed as mg/g of propolis).

		RT [min]	Uv max [nm]	[M−H]^−^	4 EtOH	4 PG	4 CC:PG (1:3)	4 CC:PG (1:4)	4 CC:Gly	5 EtOH	5 PG	5 CC:PG (1:3)	5 CC:PG (1:4)	5 CC:Gly
Phenolic acid and benzene derivatives														
1	Caffeic acid	14.34	323	179.0346	2.7	1.9	1.9	1.9	1.6	4.3	3.1	3.2	3.3	2.9
2	Vanillin	14.85	309, 280	151.0402	3.9	4.4	5.7	5.5	3.4	5.7	5.6	6.0	5.7	4.8
3	*p*-Coumaric acid	15.76	310	163.0405	18.8	16.0	17.0	15.8	13.0	24.3	19.3	19.5	20.4	17.6
4	Ferulic acid	16.46	325	193.0511	1.4	1.2	1.3	1.2	1.0	2.3	1.9	1.8	1.9	1.6
5	Isoferulic acid	16.92	324	193.0503	1.4	1.2	1.2	1.2	1.0	1.8	1.4	1.5	1.5	1.3
6	Caffeic acid ethyl ester^a^	20.99	321	207.0663	1.6	1.4	1.3	1.3	1.1	1.3	1.0	1.0	1.1	0.9
7	1,3-di-*p*-Coumaroylglycerol^b^	28.43	312	193.0505	1.3	1.0	1.1	1.0	0.8	3.4	2.6	2.5	2.7	2.6
8	Caffeic acid 2-methyl-2-butenyl ester^a^	31.42	325	247.0981	1.6	1.3	1.3	1.3	1.2	3.5	3.0	2.6	2.8	2.4
9	Caffeic acid 3-methyl-2-butenyl ester^a^	32.12	325	247.0981	1.3	1.2	1.2	1.2	1.0	6.6	5.5	5.0	5.2	4.5
10	Caffeic acid phenethyl ester (CAPE)^a^	35.25	326	283.0977	3.1	2.7	2.7	2.6	2.3	8.8	7.5	7.1	7.4	6.1
1 1	2-Acetyl-1,3-di-*p*-coumaroylglycerol^b^	36.08	312	425.1253	0.6	0.5	0.5	0.4	0.2	5.9	5.0	4.5	5.2	3.3
12	(R/S) 2-Acetyl-3-*p*-coumaroyl-1-feruloylglycerol^b^	36.39	316	455.1336	4.4	3.8	4.0	3.7	2.7	7.5	6.5	6.7	6.8	4.4
13	*p*-Coumaric acid 3-methyl-3-butenyl ester^b^	37.83	313	231.1032	17.1	14.9	15.7	14.7	10.7	21.3	16.8	16.9	17.7	12.2
14	*p*-Coumaric acid benzyl ester^b^	38.02	312	253.0878	11.6	10.3	10.6	9.9	7.5	18.4	14.2	14.2	14.9	6.6
15	*p*-Coumaric acid phenethyl ester^b^	40.14	310	267.1032	11.4	9.9	10.3	9.6	6.3	18.0	12.5	12.8	13.4	8.4
16	*p*-Coumaric acid cinnamyl ester^b^	41.96	313	279.1033	12.6	11.4	11.7	11.1	8.3	19.8	15.9	16.3	16.9	12.2
		Sum			94.6	83.1	87.6	82.5	62.1	153.0	121.9	121.6	127.0	91.8
Flvonoids and chalcones														
17	Pinobanksin 5-methylether^c^	25.29	287	285.0772	2.4	2.1	2.2	1.9	1.6	6.4	4.8	4.9	5.1	4.3
18	Pinobanksin	26.63	292	271.0617	6.6	5.7	5.8	6.3	5.0	10.4	9.8	9.8	9.7	8.7
19	Apigenin	27.36	335, 290sh, 267	269.0459	1.4	1.1	1.2	1.1	0.8	3.3	2.4	2.4	2.6	1.9
20	Kaempferol	27.65	366, 295sh, 265	285.0409	3.3	2.7	2.7	3.3	2.0	4.8	3.7	3.8	3.9	3.3
21	Chrysin	33.24	268, 312sh	253.0511	8.7	7.4	7.4	7.1	5.2	24.0	17.7	18.1	19.1	13.3
22	Pinocembrin	33.99	290	255.0668	15.4	13.4	13.7	13.1	10.8	29.7	25.9	23.4	27.9	20.2
23	Sakuranetin	34.34	290	285.0773	7.4	6.3	6.8	6.3	4.8	11.5	8.7	8.9	9.5	7.2
24	Galangin	34.72	358, 266	269.0461	14.5	10.2	11.3	9.5	8.2	23.4	18.6	18.7	19.7	15.5
25	Pinobanksin 3-*O*-acetate^c^	35.84	295	313.0725	13.3	11.6	11.6	11.3	8.9	31.5	24.5	24.1	25.7	20.0
26	Pinobanksin-3-*O*-propanoate^c^	39.62	295	327.0879	8.3	7.7	7.9	7.5	5.0	19.2	13.5	13.0	14.7	10.2
27	Pinostrobin chalcone^d^	40.56	343	269.0814	tr	tr	tr	tr	tr	3.6	3.1	2.7	2.8	2.0
28	2′-6′-Dihydroxy-4′-methoxydihydrochalcone^e^	41.18	286	271.0978	4.9	4.5	4.7	4.4	2.9	11.7	8.1	7.8	8.9	6.1
29	Pinostrobin	42.25	290	-	15.8	14.3	14.2	13.5	6.6	31.1	23.1	22.3	24.4	13.9
30	Pinobanksin 3-*O*-pentenoate or isopentenoate isomer^c^	42.90	292	353.1035	9.5	8.3	8.7	8.2	1.4	18.1	13.1	11.2	14.2	2.1
31	Pinobanksin 3-*O*-pentanoate or isopentenoate isomer^c^	45.08	291	353.1034	1.5	1.3	1.3	1.3	0.3	4.8	3.2	3.0	2.1	1.5
32	Pinobanksin 3-*O*-hexanoate or isohexanoate isomer^c^	46.49	292	367.1189	2.0	1.8	1.4	1.5	0.2	20.4	11.0	6.0	10.3	0.6
		Sum			114.9	98.4	100.7	96.2	63.7	253.9	191.1	180.0	200.7	130.8

*tr* traces.

^a^Calculated as caffeic acid.

^b^Calculated as *p*-coumaric acid.

^c^Calculated as pinobanksin.

^d^Calculated as pinocembrin chalcone.

^e^Calculated as pinocembrin dihydrochalcone (in the case of the determination per related compound, the result was corrected on the basis of the exact molecular weight).

The total amount of the quantified major compounds in different propolis extracts ranged from 125.8 to 209.5 mg/g propolis (sample 4) and 222.6 to 406.8 mg/g propolis (sample 5). In both cases, the EtOH extract contained the highest amounts of compounds while CC:Gly extract contained the lowest amounts (nearly two times less). This result explains higher MIC values of the extracts produced with CC:Gly (Table 7) used as a solvent. As mentioned above surprisingly high antimicrobial efficiency of this extract in the kill-time assay performed for *C. glabrata* is probably a consequence of using higher final concentration of this product(Fig. 3). Considering individual ingredients CC:Gly extract contained importantly lower concentration of *p*-coumaric acid derivatives (among phenolic acid and benzene derivatives) and some pinobanksin derivatives (among flavonoids and chalcones) compared to extract produced with other solvents, particularly EtOH. Other extracts (PG, CC:PG (1:3), CC:PG (1:4)) were comparable with EtOH and contained 74.1–89.9% of the content in EtOH extracts. This result finally confirms that these three NADES and also PG can be considered as alternatives for ethanol as a solvent for extraction for polish poplar propolis.

However, there were more notable differences in the extraction of individual compounds in favour of EtOH, particularly in the case of chrysin, galangin and caffeic acid. On the other hand, vanillin was extracted better by PG, CC:PG (1:3) and CC:PG (1:4) than by EtOH.

## Conclusion

The outcomes of our study revealed that NADES can be considered as an ethanol alternative for preparing propolis extracts. Among eight studied NADES, compositions of choline chloride and propylene glycol (CC:PG(1:3) and CC:PG(1:4)) have been found to be as most suitable alternative extraction solvents. The research in this area should be continued. Many other options for preparing NADES are available and we believe that some of them will enable the preparation of extracts with high health-promoting potential (including antimicrobial activity) and safe for patients.

### Supplementary Information

Supplementary Figures.

## Data Availability

All data generated or analysed during this study are included in this published article and its supplementary information files.
