# Supplementary material for: Investigation of antifungal and antibacterial potential of green extracts of propolis
Source: Sci Rep. 2024 Jun 13;14:13613. doi: 10.1038/s41598-024-64111-7 (PMC11176312; doi:10.1038/s41598-024-64111-7)

**Investigation of Antifungal and Antibacterial Potential of**

**Green Extracts of Propolis**

Jeslin Cheruvathoor Jenny^a^, Piotr Marek Kuś^b^, , Piotr Szweda^a^,*

a Department of Pharmaceutical Technology and Biochemistry, Faculty of Chemistry, Gdańsk University of Technology, ul. G. Narutowicza 11/12, 80-233 Gdańsk, Poland

b Department of Pharmacognosy and Herbal Medicines, Wrocław Medical University, ul. Borowska 211a, 50-556 Wrocław, Poland

* - corresponding author – pioszwed@pg.edu.pl, Department of Pharmaceutical Technology and Biochemistry, Faculty of Chemistry, Gdańsk University of Technology, ul. G. Narutowicza 11/12, 80-233 Gdańsk, Poland


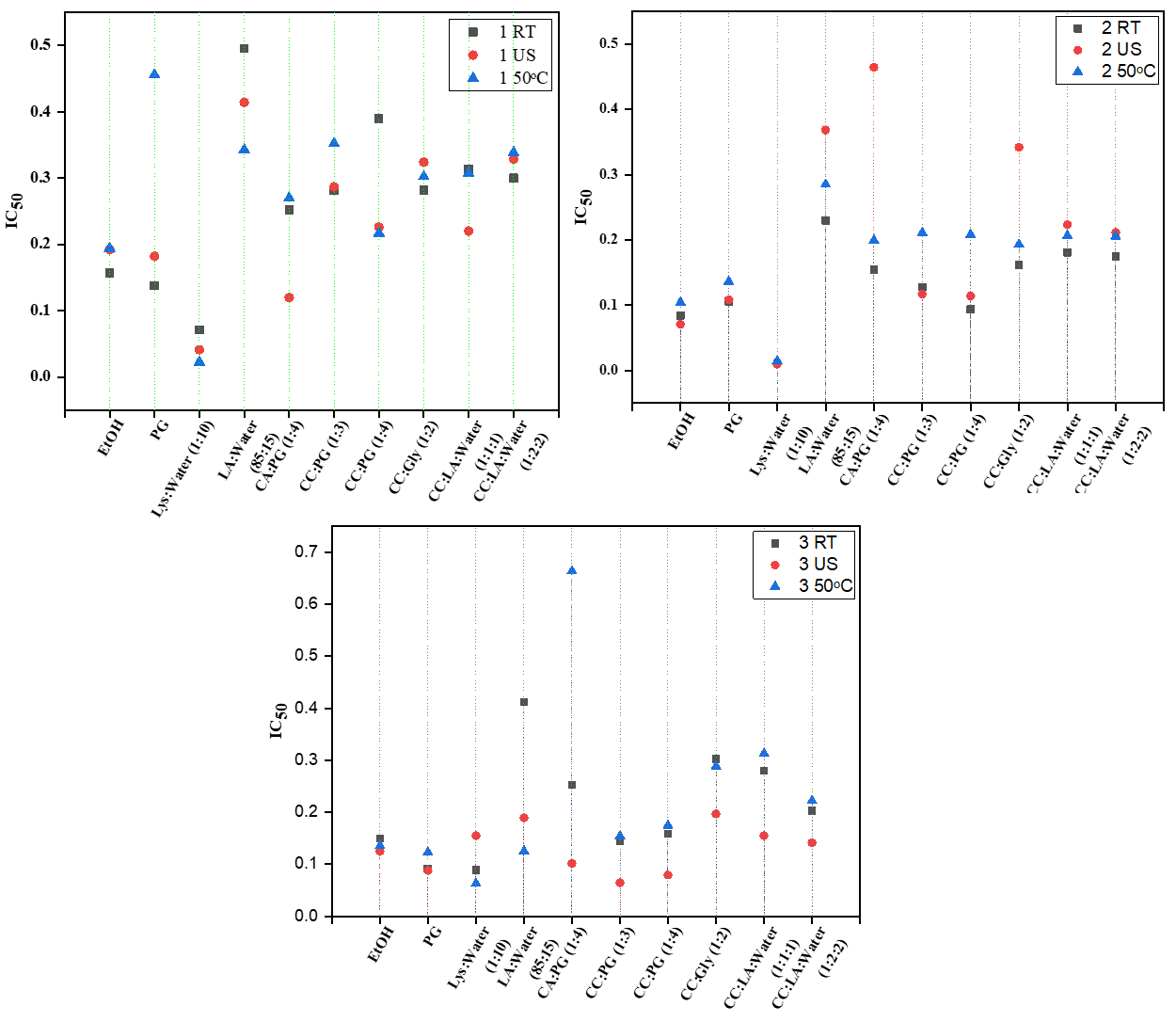


**Figure S1:** Results of DPPH assay. IC_50_ values (in (v/v) %) of propolis extracts 1, 2 and 3 obtained at room temperature (RT), by ultrasonication (US) and at 50ᵒC (50ᵒC) represented as mean ± SD.

**Figure S2**: HPLC chromatograms (a) sample 4 and (b) sample 5 extracted in EtOH, PG, CC:PG(1:3), CC:PG(1:4) and CC:Gly


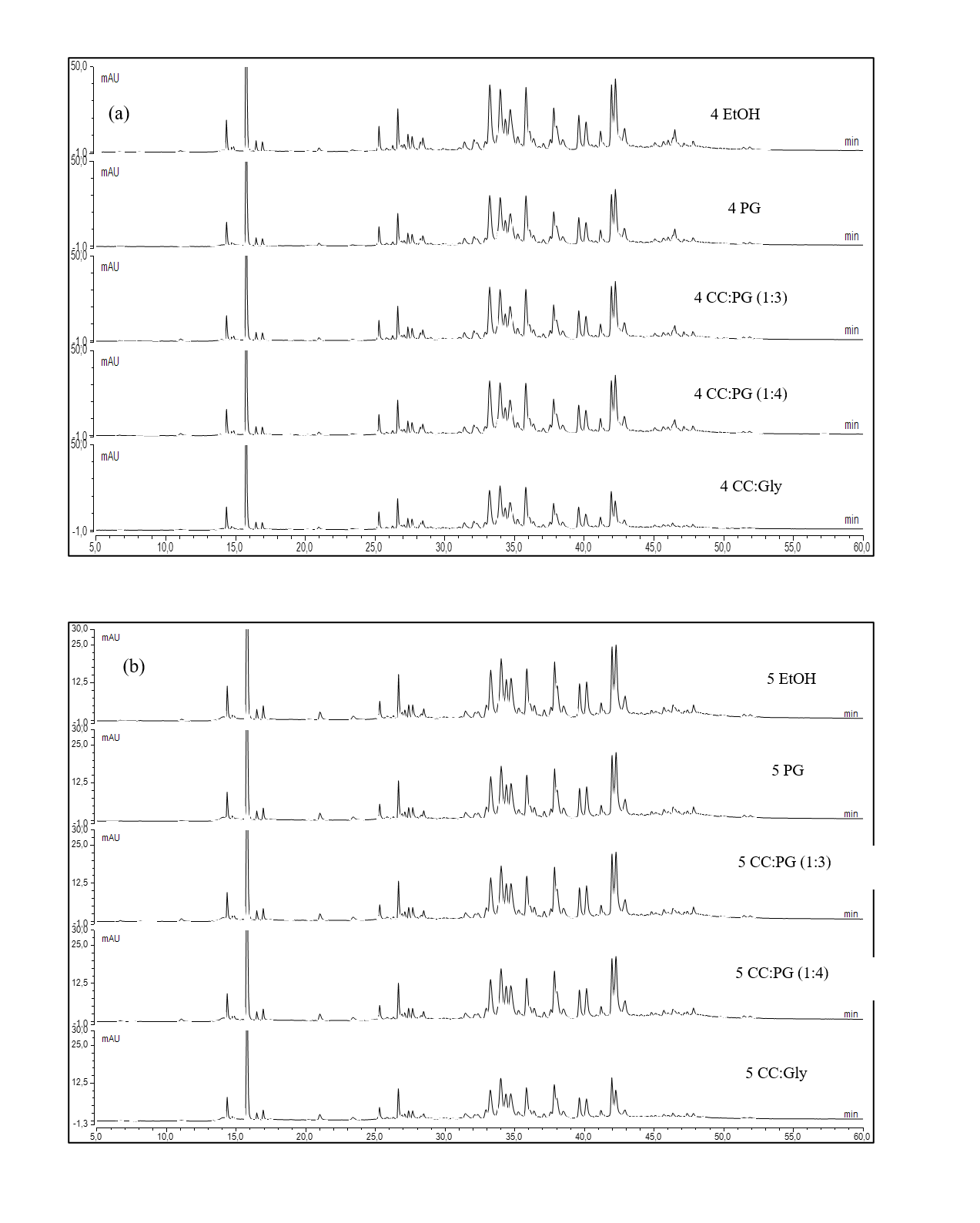

Supplement: Supplementary file 1 — Supplementary Figures. [file 41598_2024_64111_MOESM1_ESM.docx]
